# *Escherichia coli* O157:H7 transcriptome datasets for comparison of RNA-seq and microarray platforms

**DOI:** 10.1016/j.dib.2018.11.136

**Published:** 2018-11-30

**Authors:** Ewa Grabowiecka, David Martin, Louise Crozier, Nicola Holden

**Affiliations:** a The University of Dundee, Dundee, UK; b James Hutton Institute, Dundee, DD2 5DA, UK

## Abstract

Whole transcriptome analysis to investigate differential gene expression and regulatory adaption can be carried out on two different technological platforms: by probe hybridisation to microarrays or by RNAseq for deep sequencing. Since there are difference in terms of their genome coverage, sensitivity and cost, there is a requirement for robust comparisons to determine the platform of choice. Here, we present datasets for the whole transcriptional response verocytoxigenic *Escherichia coli* (VTEC) obtained from RNA-seq and microarray platforms in response to spinach, together with a comparison between the datasets (available at Array Express: E-MTAB-3249, E-MTAB-4120, E-MTAB-7441).

**Specifications table**

**Table:** 

Subject area	Biology
More specific subject area	Bioinformatics; Microbiology
Type of data	Table, graph, figure
How data were acquired	High-throughput RNA-sequencing; Microarray
Data format	Filtered and analysed with statistical tests
Experimental factors	*E. coli* O157:H7 was grown in minimal medium at 18 °C to mid-log phase and transferred to medium containing 40% (v/v) spinach leaf lysate for 1 h
Experimental features	Total RNA was extracted using commercial kits and a cDNA library generated with enterobacteria-specific primers and hybridized to a microarray (E. coli v2 array – Agilent), or rRNA was depleted and paired-end cDNA libraries generated for sequence on an Illumina Hi-Seq. 2000. A series of statistical analyses was used for comparison between the datasets.
Data source location	James Hutton Institute, Dundee, DD2 5DA, UK.
Data accessibility	Data are with this article and also available at ArrayExpress:
	E-MTAB-3249 (microarray) https://www.ebi.ac.uk/arrayexpress/experiments/E-MTAB-3249/
	E-MTAB-4120 (microarray) https://www.ebi.ac.uk/arrayexpress/experiments/E-MTAB-4120/
	E-MTAB-7441 (RNAseq) https://www.ebi.ac.uk/arrayexpress/experiments/E-MTAB-7441
	Scripts used for data analysis are available on GitHub: https://github.com/TheMicroGirl/SakaiRNASeq
Related research article	L. Crozier, P. Hedley, J. Morris, C. Wagstaff, S.C. Andrews, I. Toth, R.W. Jackson, N. Holden, Whole-transcriptome analysis of verocytotoxigenic *Escherichia coli* O157:H7 (Sakai) suggests plant-species-specific metabolic responses on exposure to spinach and lettuce extracts, Front Microbiol, 7, 2016. doi: 10.3389/fmicb.2016.01088[1]

**Value of the data**

• Direct comparison between transcriptome platforms can allow for the optimal approach to be chosen.
• Microarray platforms can offer a cheap and easy approach for transcriptional analysis for model organisms, like *Escherichia coli* O157:H7, but are limited by the probe set and potentially, sensitivity. RNA-seq does not have the same limitations but is costlier and requires specific skills for analysis.
• There are few published reports that make direct comparisons of the platforms; most adopt either one or the other.
• Here, the same sample set was applied to RNAseq and microarray transcriptome platforms to provide the most robust comparison.
• The comparison of the datasets showed a strong correlation between the platforms (*R* > 0.8) but the presence of outliers highlights differences in their outputs.

## 1 Data

**Fig. 1:**
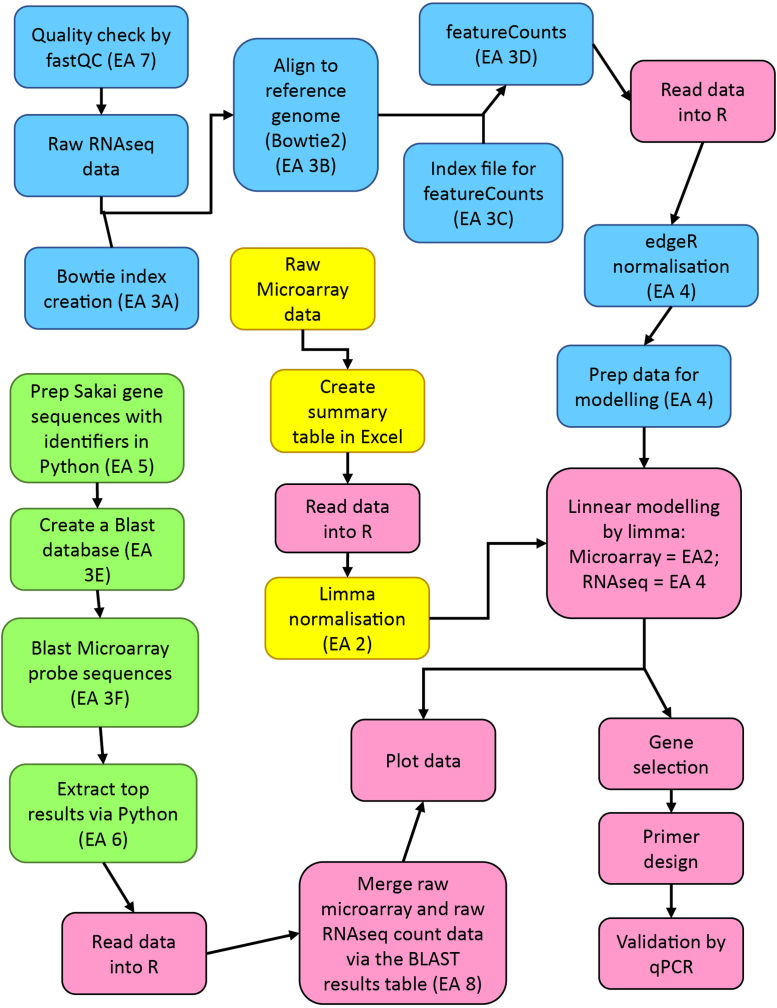
Flow diagram of analysis steps and processes. RNAseq (blue) and microarray (green) process steps are in blue and green respectively. Other steps were done in R (pink) or externally (yellow).

**Fig. 2:**
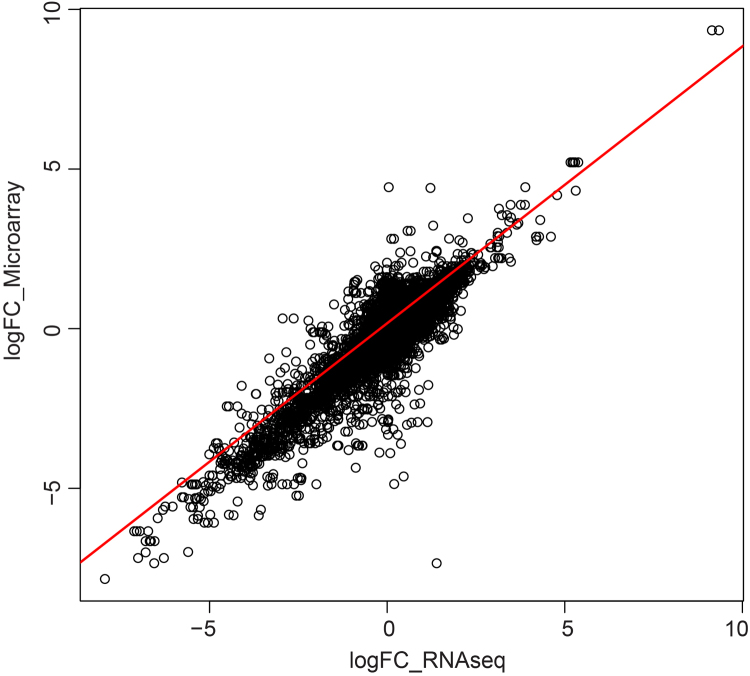
Correlation graph of expression profiles. Correlation of differential expression of genes by Log fold-change (logFC), acquired from the RNAseq and microarray datasets.

The microarray [1] and RNA-seq datasets are available in ArrayExpress. A comparative analysis pipeline (Fig. 1) was implemented for bioinformatics analysis and downstream assessment. The microarray dataset required additional processing steps since it is based on probes rather than genes and contains non-target probes from multiple *Escherichia coli* isolates. Correlation between the datasets from each platform required normalization before the comparison of the Log-fold change (spinach leaf lysate condition relative to the control no-plant condition) could be made. The Pearson and Kolmogorov–Smirnov tests of the Log-fold change datasets indicated strong correlation, although the distribution was tailed outside the range of −2 to +2-fold change (Fig. 2).

## 2 Experimental design, materials and methods

### 2.1 Sample preparations

RNA and cDNA samples from the *E. coli* O157:H7 Sakai strain were obtained and used for microarray analysis as described previously [1]. The cDNA library for RNA-Seq was constructed from the same RNA samples, using the approach described for previously *Xanthomonas* [2], and run on an Illumina Hiseq. 2000 (Genomic Sequencing Unit, the University of Dundee, Dundee, UK).

### 2.2 Data analysis

All bioinformatics scripts and processes are listed in Table 1 and illustrated in Fig. 1.

**Table 1:** Analysis steps (EA) and associated scripts and processes.

**EA #**	**Description**	**Scripts (refer to GitHub) and codes**
1	Process flow diagram	n/a: graphics (Fig. 1)
2	Script for microarray analysis	1.Microarray_Data_Analysis
3A	Script for generating the Bowtie2 index	Bowtie2-build –f sakaigenome.fasta EcoliSakai
3B	Script for both paired end and unpaired alignment using Bowtie2	Unpaired: bowtie2 -N -x EcoliSakai -U file1.fastq.gz, file2.fastq.gz.sam file
		Paired end: bowtie2 -N -x EcoliSakai -1 file.fastq.gz -2 file2.fastq.gz –S.sam file
3C	Script for index of featureCounts	{ printf ׳GeneID/tChr/tStart/tEnd/tStrand/n׳; (grep -v CDS Sakai.gff | grep gene| sed -e ׳s/[^/t]*gene=//׳ -e ׳s//.[0–9]//׳| awk ׳BEGIN{OFS="/t"}{print $9, $1, $4, $5,$7}׳)} |uniq > exons.saf
3D	featureCounts script	featureCounts –a exons.saf –F SAF –o outputfile.txt.sam file
3E	Script for using blastdb for creation of Sakai database	formatdb name.fasta –n databaseName –t title –p F
3F	Script for using matching the probe sequences to the database	blastall -p blastn –m8 –I Agilentprobes.fasta –d SakaiDatabase –v |-o output.txt
4	Script for RNA-Seq analysis	2.RNASeq_Data_Analysis/3. DEG_analysis
5	Python Script for changing sequence headers in a fasta file	3.Microarray_Vs_RNASeq/1.fasta_file
6	Pyhon Script for removing blast hits which are not suitable	3.Microarray_Vs_RNASeq/2.blastn and sort
7	FastQC reports	2.RNASeq_Data_Analysis/1.FastQC
8	Generation of Volcano Plots	Volcano plot
9	Microarray Top Table	1.Microarray_Data_Analysis
10	RNASeq Top Table	2.RNASeq_Data_Analysis/3. DEG_analysis
11	Comparison between RNAseq samples for paired end and random alignment	2.RNASeq_Data_Analysis/2.Alighment_and_Count
12	Comparison between Microarray and RNASeq	3.Microarray_Vs_RNASeq/3.table merge

#### 2.2.1 Microarray

The published microarray dataset [1] was reanalysed to permit a dataset comparison. Raw data were normalised between samples to ensure consistency across data sets. Probes from each sample were filtered in accordance with the corresponding fluorescence of the negative control probes on the microarray plate. Only probes which were 10% brighter than the 95% percentile of the negative probes were maintained for the analysis. Linear modelling was applied to the data using the R (v3.0.2) statistical language in the R studio software (v0.97.551) [3] utilising the Bioconductor Limma library (v3.18.7) [4] and following the Limma user guide (Script EA 2 – Table 1). A dendrogram was produced using the gPlots 2.12.1 [5].

#### 2.2.2 RNA-seq

FastQC software (v0.10.1) [6] was used to perform a quality check of the raw data, according to the software specifications. The reads were then aligned to the Sakai reference genome (GenBank accession number BA000007) using the Bowtie (v2 2.1.0) aligner [7]. Firstly, an index was built using the Index Builder provided by Bowtie2 (Script EA 3A) and this was then used to align the RNA samples to the reference genome (Script EA 3B). Paired-end and random alignment was performed to compare the two (Script EA 11), producing one.sam file per each sample. Reads were then summarised using the featureCount software [8]. First, an annotation file was generated using the Sakai reference genome (GenBank accession number BA000007) (Script EA 3C). The generated reference file was then used to extract the count information from the aligned.sam files (Script EA 3D). The output was a tab delimited text file containing the name of the gene, start and end positions of the gene on the strand, count of mRNA and the strand direction. Files were then imported into R studio, normalised, and fitted into a linear model using the Voom function from the Bioconductor libraries Limma [9] and edgeR (v3.2.4) [10]. The log of the fold change between experiment and control cultures was obtained from this linear model (Script EA 4). Volcano plots were generated (Script EA 12) using the ggplots2 package [11].

#### 2.2.3 Dataset comparisons

*E. coli* O157:H7 Sakai sequences, containing gene identifiers, were downloaded from the NCBI database (Sakai: NC_002695.1, pOSAK1: NC_002127.1, pO157: NC_002128.1) and converted into the appropriate fasta format using the Sequence Format Converter. A Python (v2.7.6) script [12] was written to convert fasta identifiers into a suitable format for the microarray against the RNA-Seq comparison (Script EA 5). The re-formatted fasta file was then used to construct a searchable database by using Blast (v2.2.17) [13] tool blastdb (Script EA 3E). The Blastall tool was used to match the microarray probe sequences to the above described database (Script EA 3F), resulting in a list of microarray to ECs number matches. The list of matches was filtered to extract only matches in which the sequence length was higher than 50 and mismatch was lower or equal to 7 (Script EA 6). Raw data and Log-transformed fold-change (logFC) values of normalised microarray and RNA-Seq data were compared and plotted using R studio (Script EA 12); (Fig. 2).
